# Protective Actions in Apical Periodontitis: The Regenerative Bioactivities Led by Mesenchymal Stem Cells

**DOI:** 10.3390/biom12121737

**Published:** 2022-11-23

**Authors:** Ping Lyu, Yiming Song, Ruiye Bi, Zucen Li, Yali Wei, Qin Huang, Chen Cui, Dongzhe Song, Xuedong Zhou, Yi Fan

**Affiliations:** 1 National Clinical Research Center for Oral Diseases, State Key Laboratory of Oral Diseases, Department of Cariology and Endodontics, West China Hospital of Stomatology, Sichuan University, Chengdu 610041, China; 2 National Clinical Research Center for Oral Diseases, State Key Laboratory of Oral Diseases, Department of Oral and Maxillofacial Surgery, West China Hospital of Stomatology, Sichuan University, Chengdu 610041, China; 3 Guangdong Province Key Laboratory of Stomatology, Hospital of Stomatology, Guanghua School of Stomatology, Sun Yat-Sen University, Guangzhou 510055, China

**Keywords:** periapical inflammation, tissue regeneration, dental stem cells, regenerative therapy

## Abstract

Resulting from bacterial infection, apical periodontitis (AP) is a common inflammatory disease of the periapical region of the tooth. The regeneration of the destroyed periapical alveolar bone and the surrounding periodontium tissues has long been a difficult task in clinical practice. These lesions are closely related to pathogen invasion and an overreactive immune response. It is worth noting that the protective healing process occurs simultaneously, in which mesenchymal stem cells (MSCs) have a crucial function in mediating the immune system and promoting regeneration. Here, we review the recent studies related to AP, with a focus on the regulatory network of MSCs. We also discuss the potential therapeutic approaches of MSCs in inflammatory diseases to provide a basis for promoting tissue regeneration and modulating inflammation in AP. A deeper understanding of the protective action of MSCs and the regulatory networks will help to delineate the underlying mechanisms of AP and pave the way for stem-cell-based regenerative medicine in the future.

## 1. Introduction

Apical periodontitis (AP) is an inflammatory disease in the periapical region of the tooth, with a high degree of morbidity. A study reported that 52% of the adult population worldwide has had at least one tooth affected by AP [1]. The progression of AP depends on the dynamic equilibrium between the invading microorganisms and host defense mechanisms. Patients suffer from acute AP when pathogens overwhelm the immune actions. The clinical symptoms of AP include severe abscess, local swelling, and fistula [2]. Inflammatory immune reactions and toxins secreted by pathogens destroy periradicular periodontium tissues [3,4]. Without clinical treatment, an AP lesion could gradually evolve and develop into a sinus tract, leading to severe bone defects. The damage of the periodontal ligament and local bone tissues results in tooth mobility and ultimately causes tooth loss. Thus, AP contributes greatly to the global burden of dental diseases [1].

Notably, protective healing bioactivities occur simultaneously with the pathological process in AP, which is mainly led by mesenchymal stem cells (MSCs), which are primitive cells characterized by self-renewal and a multilineage differentiation potential. Physiological MSC clusters derived from the dental region include orofacial bone-marrow-derived mesenchymal stem cells (OMSCs), periodontal ligament stem cells (PDLSCs), dental pulp stem cells (DPSCs), stem cells from human exfoliated deciduous teeth (SHEDs), gingival mesenchymal stem cells (GMSCs), the stem cells from the apical papilla (SCAPs), and dental follicle progenitor cells (DFPCs) [5,6]. Among them, OMSCs and SCAPs have been demonstrated as critical participants in the repair process in AP [7,8,9,10,11,12]. Research applying dental MSCs in vivo has shown their potential in the formation of bony tissues and a root–periodontal-like complex as well as pulp regeneration [5].

In 2004, fibroblastic cells with an osteogenic ability were discovered and isolated from inflamed human periapical granulation tissue [13]. These cells were able to differentiate into osteoblastic or cementum lineage cells and generate a calcified matrix in vitro, suggesting their potential in periapical osseous healing. In 2013, Marrelli et al., isolated and characterized MSCs from human periapical cysts and termed them human periapical cyst–mesenchymal stem cells (hPCy–MSCs) [14,15]. These cells are another promising candidate for tissue engineering and a therapeutic target for AP. Moreover, MSCs can exert an immunomodulatory effect to promote tissue regeneration. MSCs can reportedly suppress apoptosis, exert an anti-inflammatory effect, enhance angiogenesis, and modulate the extracellular matrix under inflammation [16]. Dental MSCs showed immunoregulatory effects in various inflammatory and autoimmune diseases, including periodontitis, Sjögren’s syndrome, colitis, and rheumatoid arthritis [6].

Given the remarkable protective and regenerative functions of MSCs, this review aims to summarize the protective bioactivities led by MSCs in AP, as well as the MSC-based therapeutic strategies. More intensive studies focusing on protective actions in AP will lead to improved MSC-based regenerative therapy for AP and other inflammatory diseases within the orofacial bone area.

## 2. The Pathogenic Factors of the Periapical Microbial Community

More than 700 species of microorganisms colonize the oral cavity and orchestrate the microenvironment. AP results from a bacterial infection through the root canal system. Dental caries, cracks, traumatic injuries, and structural defects create invasive routes for bacteria [17]. Deep periodontal pockets and gingival sulci can also provide infectious pathways through the periodontium blood supply. The most common cause of AP is tooth decay, the development of which gradually leads to pulpitis and ends with pulp necrosis and AP [18]. The microbial community in AP was studied through culture or molecular methods. Samples were collected through the root canals of AP-affected teeth and the purulent exudate from the fistula. Although the most prevalent species vary from study to study, the microbial diversity in an apical abscess is dominated by anaerobic bacteria. The most frequently detected bacterial species belong to seven bacterial phyla: *Firmicutes*, *Bacteroidetes*, *Fusobacteria*, *Actinobacteria*, *Spirochaetes*, *Synergistetes*, and *Proteobacteria*. Among these, the *Firmicutes* and *Bacteroidetes* phyla account for over 70% of the species discovered in the abscesses [19]. The pathogenic factors of bacteria include structural components and harmful products of bacterial metabolism. The former can activate the immune system, while the latter directly damages periapical cells and tissues. Lipopolysaccharides (LPS) are a major component of the cell wall of Gram-negative bacteria. LPS plays a primary role in the progression of AP and the acute immune response. It activates various immunocytes and stimulates the release of a series of pro-inflammatory factors, which result in inflammatory infiltration and bone destruction. Moreover, LPS can directly stimulate osteoclast formation and enhance bone resorption by stimulating osteoblasts to secrete the receptor activator of the nuclear factor kappa B ligand (RANKL). It is also crucial in triggering early inflammation and the whole immune response by inducing the expression of leukocyte adhesion molecules in endothelial cells, activating the complement system and B cells, and evoking pain. Lipoteichoic acid (LTA) is a structural virulence factor of Gram-positive bacteria, endowing the bacteria with adhesive properties associated with medication resistance and biofilm formation. LTA can also activate leukocytes, macrophages, and monocyte populations to release immune mediators [18].

## 3. Components Related to Inflammatory Bone Loss

The apical bone lesion in AP results mainly from the infiltration of immune cells and the secreted molecules. Persistent inflammation activates osteoclasts and results in inflammatory bone resorption [20]. In this section, we briefly introduce the main regulator of osteoclast formation and the activities of immunocytes related to bone resorption.

### 3.1. RANKL/RANK/OPG System

The RANKL/RANK/OPG system leads the bone resorption process since it controls the formation, maturation, and activities of osteoclasts [21]. RANKL is a TNF family member produced by bone marrow stromal cells, osteoblasts, osteocytes, and immune cells. It can activate the receptor activator of NF-κB (RANK) of the osteoclast precursor and mature osteoclasts to support bone resorption activities. Osteoprotegerin (OPG) is a soluble decoy receptor for RANKL mainly secreted by osteoblasts. It has a higher affinity than RANK and, therefore, inhibits the RANKL–RANK interaction [22]. The RANKL/OPG ratio is an effective indicator of bone remodeling activities, including bone formation and resorption. A diverse change in the RANKL/OPG ratio can determine the stability of an AP lesion [23]. Research shows that increased bone resorption in the case of an AP lesion is accompanied by a high RANKL/OPG ratio. Contrarily, a low RANKL/OPG ratio along with arrested osteoclast activity resulted from the steady immunoreactions [24].

### 3.2. Neutrophils

As the most abundant immunocytes in the circulatory system, neutrophils are the first-line responders in the case of infection and tissue injury [25]. The immediate response of neutrophils is of great importance in the defense against pathogens and the progression of inflammatory lesions in AP [26]. Neutrophils release various antimicrobial substances, including reactive oxygen species (ROS), proteases, and toxic enzymes, to debride pathogens and devitalize tissues [27]. Therefore, inflammatory infiltration and tissue damage occur simultaneously. Mediators secreted by neutrophils function as chemotaxis to recruit other immune cells, including macrophages and lymphocytes [28]. Stimulated by LPS, neutrophils produce interleukin-1 (IL-1) and IL-6 to auto-stimulate a neutrophil oxidative burst and cytotoxicity. These two molecules also contribute to the activation and maturation of osteoclasts, leading to bone resorption. AP patients with severe bone loss and fistula reportedly showed a higher expression of IL-17 [29]. IL-17 presented the ability to recruit large groups of neutrophils, enlarging the bone lesion [30].

### 3.3. Macrophages

Belonging to the family of phagocytes, macrophages are another innate immune participant in protective activities against AP. Macrophages help remove pathogenic microorganisms, toxins, other foreign substances, and dead cells [31]. Notably, macrophages are able to produce both pro- and anti-inflammatory biomolecules to initiate and regulate the AP process. Cytokines, including tumor necrosis factor α (TNF-α), IL-1α, IL-6, and prostaglandins, recruit and differentiate osteoclasts. It is reported that prostaglandins synthesized by macrophages modulate the functions of osteoclasts and the resorptive intensity in AP [32]. These inflammatory molecules and transforming growth factor β (TGF-β) lead to neo-vascularization, fibroblast activation, and collagen secretion, thus forming the granulation tissues.

### 3.4. T Cells

The T lymphocyte family is composed of Th1, Th2, Th17, regulatory T cells (Tregs), and other subclusters [33]. The differential fate of T cells is mainly directed by the receiving peptides from antigen-presenting cells [34]. Different subtypes of T cells were identified by their pattern of cytokine secretion [35]. Th1 cells are believed to play a pro-inflammatory role through Th1-related cytokines, including IL-2, IL-12, and interferon-gamma (IFN-γ) [36]. Th2 usually exerts an immunosuppressive function, the related cytokines of which include IL-4, IL-5, and IL-13 [33,37,38]. Interestingly, only IFN-γ-positive T cells were detected in periapical infiltrated tissues, while IL-4-positive T cells were observed in the regenerative area [39]. Moreover, higher IFN-γ expression was detected in human AP lesion samples with a high RANKL/OPG ratio. Higher levels of Th2-specific cytokines, including IL-4 and IL-33, were detected in samples with a low RANKL/OPG ratio [40]. These findings indicate that periapical lesion regeneration requires proper bone remodeling and a suitable immune microenvironment related to Th2 cells. Th17 cells are another key component contributing to AP progression [41,42]. They can produce IL-17 to recruit the neutrophil population and expand the bone lesion. However, the precise function of IL-17 remains unclear since the protective function of IL-17 in AP has also been demonstrated [43]. Further research is required for a deeper understanding of the function of Th17 cells and IL-17 in AP. Tregs are characterized by CD4, CD25 double positive with the unique marker, and forkhead transcription factor P3 (Foxp3). They can suppress the T cell response and restrict the progression of lesions [35]. Research shows that Tregs secrete IL-10 and TGF-β to control local inflammation in human periapical lesions, which is probably related to the regenerative process [44].

### 3.5. Mast Cells

**Figure 1:**
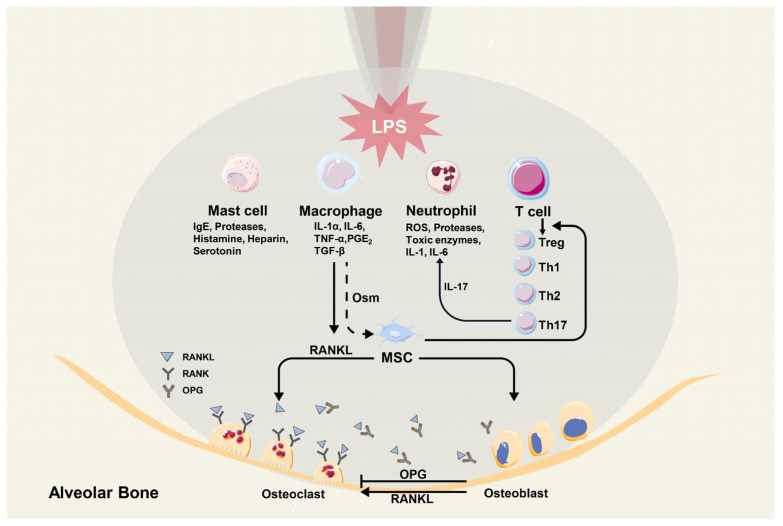
The immuno-microenvironment in a periapical lesion.

Compared with samples from radicular cysts and residual radicular cysts, more tryptase-positive mast cells were detected in tissues of periapical granulomas. Through degranulation, mast cells can release mediators, including IgE, histamine, serotonin, heparin, and several proteases, which function in fibrosis, connective tissue remodeling, and the enlargement of AP lesions [45]. Figure 1 illustrates the immuno-microenvironment of an apical lesion.

## 4. The Regulatory Network of Dental MSCs in AP

Apart from immune cells, dental MSCs play a crucial protective role in AP. Since fibroblastic cells with an osteogenic potential have been derived from periapical lesions, a number of studies have supplied evidence regarding the involvement of MSCs in AP. Markers of MSCs, including CD44, CD73, CD90, CD105, CD146, and STRO-1, have been identified in both primary and persistent AP lesions [46,47,48]. Moreover, cells isolated from inflamed periapical tissues present the ability to form colony-forming-unit fibroblasts (CFU-Fs), which is a typical feature of MSCs. Compared with normal periapical bone tissue, AP lesions expressed higher levels of STRO-1, CD44, and proliferating cell nuclear antigen (PCNA), suggesting that an inflammatory microenvironment activates MSC proliferation [49]. These cells were highly osteogenic, indicating their repair and regeneration ability [48]. The isolated hPCy–MSCs were characterized as CD73^+^, CD90^+^, CD105^+^, CD13^+^, CD29^+^, CD44^+^, CD45^−^, STRO-1^+^, and CD146^+^ using flow cytometry and immunofluorescence [14]. These cells possess the potential for in vitro osteogenesis, adipogenesis, and neurogenesis [14,15]. CD146 expression influences the properties of hPCy–MSCs. Cell populations with a low CD146 expression displayed a more potent proliferative, self-renewal ability and osteogenic potential. In some in vitro experiments, the expression of CD146 gradually decreased during passaging [50]. Notably, under the same culture conditions, hPCy–MSCs tended to be directed toward an osteogenic lineage, while DPSCs preferably differentiated into odontoblasts [51]. This finding suggests that hPCy–MSCs are better candidates than DPSCs in MSC-based regenerative therapy for bone defects.

The pro-healing mechanisms of MSCs in the case of AP are associated with their immunosuppressive capacity, which attenuates host tissue injury resulting from destructive immunoreactions [4]. MSCs isolated from periapical lesions (PL-MSCs) were able to suppress both peripheral blood mononuclear cells and local periapical inflammatory cells. When peripheral blood mononuclear cells were co-cultured with PL-MSCs, the proliferation and expression of IL-2, IL-5, and IFN-γ in the peripheral blood mononuclear cells were inhibited in a TGF-β-dependent manner. PL-MSCs also reduced IL-1β, IL-6, IL-8, and TNF-α production by periapical inflammatory cells via soluble mediators or cell-to-cell contact [52]. Araujo-Pires et al. inhibited the recruitment and mobilization of MSCs by applying a CXCR4 antagonist in mice with periapical lesions. They found that CXCR4 inhibition resulted in increased periapical lesion sizes and decreased expression of MSCs markers. Furthermore, the inhibition of MSC recruitment led to the downregulated expression of wound healing and anti-inflammatory markers as well as an upregulated expression of osteoclastogenic and pro-inflammatory cytokines, including IL-1β, IL-17, TNF-α, IFN-γ, and RANKL. These results highlight the immunosuppressive and pro-healing functions of MSCs in periapical granuloma pathogenesis [53]. In addition, periapical-lesion-derived MSCs were able to interfere with the differentiation of monocyte-derived dendritic cells (DCs). The developing DCs co-cultured with AP-derived MSCs showed poor allostimulatory activities [54]. The immunoregulatory effects of MSCs are believed to be governed by their microenvironment, especially the surrounding inflammatory intensity [55]. MSCs reportedly promoted immunoreactions under low inflammatory conditions, while they switched to anti-inflammatory cells to avoid the overexpression of immunoactivities [56]. Considering the variation in the condition and progression of AP in patients, the role of MSCs in different stages requires further elucidation.

There is evidence that dental MSCs also participate in driving AP progress and the transformation of different stages. Estrela et al. reported that the expression of Sox2, a marker for progenitor stem cells, was higher in primary periapical lesions with acute inflammatory infiltration compared with that in CD90. The latter was rich in persistent apical periodontitis with chronic inflammatory cell infiltration [47]. The histopathological diagnosis of chronic AP includes periapical cyst, abscess, and granuloma. The microenvironment of chronic AP might be associated with different stem cell lineages. MSCs positive for CD44 and CD73 have been observed in the rims of abscesses and periapical cysts [46]. Compared with their expression levels in cysts and granulomas, the expression levels of MSC markers were higher in the apical abscess, with a significant association between them [46].

AP is a complex disease with both acute and chronic phases and several histopathological types. To better understand the function and action of MSCs in the case of AP, research focusing on specific lineages of MSCs was carried out. Since 2000, various clusters of dental MSCs have been derived from dental pulp, human-exfoliated deciduous teeth, periodontal ligament, dental follicle, apical papilla, and alveolar bone [57,58,59,60,61,62]. It is worth further exploring how these cells react to stimulation from an immune response and periapical destruction.

### 4.1. Orofacial Bone/Bone-Marrow-Derived MSCs (OMSCs)

In addition to the long bone and the iliac bone, the orofacial bone is an important source of bone-marrow-derived MSCs, called OMSCs [62]. During AP progression, the inflammatory microenvironment can stimulate OMSCs. Our previous study applied the lineage tracing method to show that AP significantly stimulates the generation of Osx^+^ mesenchymal progenitors and recruits them to the lesion. We established transgenic mouse models to unravel the regulatory role of Klotho (KL), an essential component of the fibroblast growth factor (FGF) pathway. The results showed that the periapical lesion area in *OsxCre;KL^fl/fl^* AP mice significantly increased because of increased inflammatory bone resorption and attenuated osteogenesis. Klotho ablation in Osx^+^ mesenchymal progenitors led to an increased osteoclast number and dental root absorption with elevated RANKL, reduced OPG expression, and a higher RANKL/OPG ratio. Lineage tracing using the Osx:GFP and tdTomato system revealed a significantly decreased number of Osx:GFP/tdTomato double-positive cells, indicating that Klotho deficiency impaired the osteogenic potential of Osx^+^ mesenchymal progenitors. RNA-seq analysis further suggested that Klotho may negatively regulate the inflammatory response since the highest expression of TNF receptor I (TNFR1) was detected in *OsxCre;KL^fl/fl^* AP mice. Conversely, overexpressed Klotho in osteoblasts resulted in significantly suppressed Tnfr1 expression and nuclear translocalization of the TNF-α-induced NF-Κb p65 subunit. Klotho-deficient osteoblasts showed a weakened ability to support osteoclast generation. This study revealed that Osx^+^ mesenchymal progenitors are actively involved in the regeneration of AP, which is under the pro-osteogenic and anti-inflammatory effects of Klotho [63]. A recent study using 10× single-cell RNA sequencing analyzed cell populations in healthy mouse mandibular alveolar bone. The results showed that the largest population interacting with MSCs was that of monocytes/macrophages. The macrophage subcluster in the alveolar bone marrow is the major group secreting oncostatin M (Osm). Osm knockout resulted in a decreased number of osteoblasts and delayed bone healing in a tibial defect model [64], which shows that Osm exerts a regulatory effect on bone homeostasis. Hence, the Osm secreted by monocyte/macrophage may modulate the fate commitment of MSCs, resulting in different biological features of bone. Considering the essential participation of macrophages in AP, the interaction between MSCs and the macrophage cluster is a key point to be studied in the future, in addition to the regulatory role of Osm in a pathological microenvironment. Since OMSCs have been discovered in the last decade, the related research is still in the early stage. Previous studies focusing on bone-marrow-derived MSCs in AP used long-bone-origin BMMSCs to perform in vitro experiments. AP results from bacterial invasion through the root canal. *Porphyromonas gingivalis* is a crucial pathogen in AP. One study [12] demonstrated that the lipopolysaccharide of *Porphyromonas gingivalis* (Pg LPS) led to osteogenic arrest in BMMSCs, which was associated with the suppression of Wnt/β-catenin signaling. In response to Pg LPS, alkaline phosphatase (ALP) activity, mineralization ability, osteocalcin, and runt-related transcription factor 2 (Runx2)/Osterix expression of BMMSCs were significantly reduced, which was partially rescued by transient Wnt3a treatment. However, prolonged Wnt3a treatment exerted reverse effects on osteogenesis, indicating the dichotomous regulatory mechanisms of the Wnt/β-catenin pathway [12]. Although *Enterococcus faecalis* is not a general pathogen for common AP lesions, it is closely related to the failure of root canal treatment and refractory AP [65,66]. It was reported that the supernatants of *E. faecalis* upregulate miR-200A-3p, which leads to the migration of BMMSCs through the forkhead box protein J1 (FOXJ1)/NFκB axis [10]. The transcription of miR-200A-3p downregulated FOXJ1, thus attenuating its suppression on NFκB. Consequently, activated NFκB enhanced the expression of downstream MMP3 and MMP13, which resulted in enhanced BMMSC migration [10]. During the pathophysiological process of AP, MSCs not only react with pathogens but also interact with osteoclasts and the immune system. Inflammatory bone loss mainly results from the imbalance between excessively active bone absorption and deficient bone formation. Xiao et al., reported that increasing levels of sphingosine kinase 1 (SPHK1) and sphingosine-1-phosphate receptor 1 (S1PR1) appeared in human AP lesions accompanying macrophage infiltration and high levels of RANKL. The number of S1PR1-RANKL double-positive cells also increased in the lesion region. The ex vivo co-culture model of macrophages and BMMSCs under LPS stimulation revealed that macrophages induced SPHK1 activity to increase the synthesis of sphingosine-1-phosphate (S1P) and then activate BMMSC S1PR1. Thus, BMMSCs produced RANKL and then enhanced osteoclastogenesis, ultimately leading to periapical bone destruction [11].

Compared with BMMSCs, OMSCs are more proliferative, with higher in vitro ALP expression and calcium accumulation [67]. Considering the unique embryonic origin and developmental pattern of orofacial bone, OMSCs could be a better target in future studies to delineate the function of tissue-specific MSCs in AP. Our previous studies discovered that Prx1, Osx, and Leptin Receptor (LepR) are important markers for OMSCs, which could be applied in lineage tracing experiments to explore the detailed mechanisms of how these specific lineage cells participate in future AP progression [63,68,69]. With the advances in the knowledge of OMSCs, related in-depth research is possible.

### 4.2. Stem Cells from Apical Papilla (SCAPs)

The apical papilla is part of the dental papilla tissue and is located at the apex of developing permanent teeth [70]. STRO-1 was the first MSC marker identified in the apical papilla, indicating the existence of SCAPs [61]. In addition, SCAPs were found to be positive for CD29, CD105, and CD24 [61]. SCAPs are highly proliferative, with odontogenic, osteogenic, adipogenic, and neurogenic potential [71,72]. The removal of the apical papilla reportedly led to the interruption of root development, indicating the pivotal role of SCAPs in this process [70]. SCAPs can also explain the continuous apexogenesis in immature permanent teeth with periapical periodontitis after endodontic treatment. They are capable of giving rise to primary odontoblasts and enhancing root maturation. These features make SCAPs promising candidates for pulp regeneration and bio-root engineering. Regenerative endodontic treatment (RET) is a crucial therapeutic option in the treatment of immature teeth with AP. Since apical papilla is essential in RET, research has focused on the regulatory mechanisms of SCAPs under inflammation.

In one study [73], cells isolated from inflamed periapical tissue and apical papilla co-expressed CD73, CD90, and CD105, implying that inflamed SCAPs could maintain their stemness. Inflamed-apical-papilla-derived cells showed an increased proliferative rate and osteogenesis/odontogenesis abilities [73]. Compared with normal SCAPs and inflamed periapical progenitor cells, inflamed SCAPs showed greater mineralization potential with a higher expression of the von Willebrand factor, and angiogenesis and pro-coagulation factor [8]. In another study, SCAPs promoted the conversion of pro-inflammatory CD4^+^CD25^−^ T cells to CD4^+^CD25^+^Foxp3^+^ Tregs. After RET was applied to an AP dog model, Foxp3^+^ Tregs were enriched in newly formed regenerative tissue, implying the potential role of SCAPs in repairing AP lesions by regulating T cells and Tregs to create a proper immune microenvironment [9]. In addition to acting as immune regulators, SCAPs can function as target cells for endodontic treatment to enhance the repair of the inflamed root in AP. Berberine (BBR), an effective constituent in herbal medicines, has been applied in the root canals of immature teeth with AP, leading to longer roots, thicker walls, and a smaller apex compared with those in teeth treated with calcium hydroxide or sterilized saline. In an in vitro study, BBR promoted the osteogenesis of SCAPs through the canonical Wnt/β-catenin pathway. It upregulated the expression and nucleus translocation of β-catenin, which upregulated the downstream Runx2 expression [7]. MicroRNAs (miRNAs) also regulate SCAPs. The research applied miRNA sequencing on human AP apical tissues and revealed that miR-10a-5p was the most significantly upregulated miRNA in AP. The overexpression of miR-10a-5p in LPS-treated SCAPs led to the downregulation of TNF-α and increased expression of IL10, indicating the anti-inflammatory and pro-healing function of the miRNA system [74]. There is evidence that SCAPs contribute greatly to the regeneration of periapical inflamed tissues after receiving endodontic treatment. However, their role and underlying mechanisms during the ongoing peri-radicular inflammatory process need to be further explored.

### 4.3. Dental Follicle Progenitor Cells (DFPCs)

The dental follicle is a loose structure surrounding the tooth germ during the developmental stage [75]. It is composed of connective tissues and is closely related to the tooth-eruption process and periodontal tissue formation [76]. A population of cells with classic MSC features was derived from the dental follicle of human third molars and defined as DFPCs [60]. They can self-renew and are highly pluripotent, being able to differentiate into osteoblasts, adipocytes, chondrocytes, neurons, periodontal ligament cells, and cementoblasts [77]. The coronal part of the dental follicle is crucial to the formation of the eruption path by regulating the genesis of osteoclasts and the bone resorption process [78,79]. The successors of AP-affected primary teeth tend to erupt early, which is related to the activities of DFPCs under the stimulation of increasing inflammatory cytokines. According to Meng et al., DFPCs treated with IL-1α showed attenuated osteogenesis and an increasing ability to promote osteoclast generation. Higher RANKL/OPG ratios were detected in DFPCs cultured with IL-1α, and the co-culture experiment showed an increasing number of TRAP-positive osteoclasts. Contrarily, the injection of an IL-1 receptor antagonist led to decreasing osteoclasts and delayed tooth eruption in vivo [80]. DFPCs also showed immunoregulatory effects, which makes them another promising candidate for RET. It is reported that the conditioned medium of DFPCs (DFPC-CM) relieved the inflammatory states of LPS-treated dental pulp cells (DPCs). The application of DFPC-CM promoted the proliferation, migration, odontogenic ability, and ectopic dentine genesis of inflammatory DPCs. The expression of inflammatory markers, including IL-1β, IL-6, and TNF-α, was decreased, while the generation of IL-4 and TGF-β was induced. Moreover, DFPC-CM ameliorates the inflamed dental pulp by reducing inflammatory cell infiltration and triggering Runx2 expression to promote the repair of injured sites [81]. In addition to the treatment of pulpitis, these results suggest the application prospects of DFPCs in AP, which requires further exploration.

**Figure 2:**
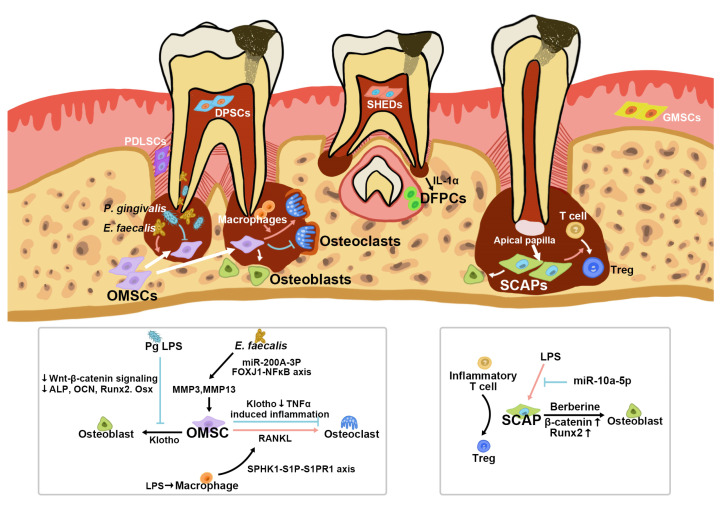
The regulatory networks of OMSCs, DFPCs and SCAPs within apical periodontitis.

AP is a complicated pathological process affecting multiple tissues, including teeth, alveolar bone, and periodontal ligament. Research focusing on the protective role of specific types of dental MSCs in AP is still in the early stages. Figure 2 illustrates the regulatory networks of OMSCs, SCAPs and DFPCs in the case of AP. Whether other populations of dental MSCs are affected by AP or contribute to the protective and healing process needs to be further elucidated. Therefore, we will also review the studies related to dental MSCs in other inflammatory diseases to inspire research focusing on AP and dental MSCs in the future.

## 5. The Immunoregulatory Functions of Other Dental MSCs

### 5.1. Dental Pulp Stem Cells (DPSCs)

As the first discovered dental MSCs, DPSCs were isolated from human dental pulp in 2000 [57]. They are highly clonogenic and proliferate rapidly. These cells express typical MSC markers, including CD44, CD90, CD105, and CD146. They also demonstrate multilineage differentiation, including osteogenic, adipogenic, neurogenic, and odontogenic abilities. In vivo transplantation showed their ability to generate dentin-pulp-like tissues, indicating their potential in dental pulp regeneration [82]. Biomaterials such as hyaluronan-based gel can also be used to enhance the osteogenic potential of DPSCs, which can be applied in the stem-cell-based treatment of bone defects [83]. DPSCs and their secreted factors are involved in the treatment of several inflammatory diseases, including pulp necrosis, periodontitis, temporomandibular joint (TMJ) arthritis, and Sjögren’s syndrome. It is reported that resolving E1 (RvE1), an omega-3 fatty acid metabolite, was applied in pulp capping to enhance the regenerative role of DPSCs in pulpitis. It promoted the recruitment of DPSCs to the damaged molar pulp in vivo. In vitro experiments showed the anti-inflammatory function of RvE1 by suppressing the secretion of pro-inflammatory cytokines, including TNF-α, IL-1β, and IL-6, while upregulating the expression of osteogenesis- and odontogenesis-related genes in LPS-treated DPSCs [84]. In addition to being the target of biomolecules to enhance the treatment of inflammatory diseases, DPSCs can be applied in regenerative medicine. Cui et al., injected DPSCs into the articular cavity of rats with TMJ arthritis, which alleviated the symptoms of pain and synovial inflammation. DPSC injection also attenuated the deterioration of the cartilage matrix, reduced the expression of inflammatory TNF-α and IFN-γ, and stimulated the bone regeneration process [85]. Moreover, the conditioned media and exosomes derived from DPSCs exerted promising immunoregulatory effects. Conditioned media from DPSCs (DPSC-CM) was applied to a Sjögren’s syndrome (SS) mouse model by intravenous injection and suppressed the activation of T cells, increased the salivary flow, and decreased the inflammation sites in submandibular glands. The results also showed that DPSC-CM administration increased the percentage of Tregs and reduced the number of Th17 cells. This study suggested that the secreted factors of DPSCs contained in the conditioned media ameliorate the inflammatory symptoms of SS by enhancing Treg generation and suppressing Th17 cell differentiation. Another study applied DPSC-derived exosomes incorporated into a chitosan hydrogel (DPSC-Exo/CS) to treat periodontitis, which ameliorated the periodontal lesions and accelerated epithelium healing. These therapeutic effects depended on the conversion of pro-inflammatory macrophages into an anti-inflammatory phenotype, related to the miR-1246 carried in DPSC-exo [86].

### 5.2. Stem Cells from Human Exfoliated Deciduous Teeth (SHEDs)

Dental MSCs were also discovered in the crown pulp tissues of exfoliated deciduous teeth, which were characterized as immature DPSCs with the expression of embryonic stem cell markers [58]. SHEDs showed a higher proliferative feature compared with adult MSCs, such as DPSCs. Their osteogenic, odontogenic, and neurogenic potential makes them a possible source of dental pulp tissue engineering and bio-root regeneration [87,88]. Carried by the treated dentin matrix, SHEDs achieved the in vivo regeneration of the periodontal ligament and the alveolar bone with a blood supply [88]. A rat periodontitis model with the local administration of SHEDs showed significantly enhanced periodontal regeneration and an increasing alveolar bone volume with a reduced distance between the cementoenamel junction and the alveolar bone crest. These therapeutic effects of SHEDs were related to the immune regulation of macrophages. Both in vivo and in vitro experiments showed that SHEDs enhance the polarization of anti-inflammatory M2 macrophages [89]. Similar to DPSCs, the transplantation of SHEDs also alleviated the symptoms of SS by promoting Treg conversion and induced apoptosis of Th17 cells via the Spd-L1/PD-1 pathway [90]. Another study used exosomes of SHEDs (SHED-exo) to treat TMJ osteoarthritis (TMJOA). SHED-exo suppressed TMJ inflammation and the expression of IL-6, IL-8, matrix metalloproteinase 1 (MMP1), MMP3, MMP9, and MMP13 via the miR-100-5p/mTOR axis.

### 5.3. Gingival Mesenchymal Stem Cells (GMSCs)

The lamina propria/connective tissue layers of gingiva contain a population of adult stem cells. They possess multilineage differential abilities, including osteogenesis, adipogenesis, and neurogenesis. GMSCs are positive for classic MSC markers, including CD73, CD90, CD105, and STRO-1 [91]. They are widely applied in the treatment of different inflammatory diseases on account of their strong immunomodulatory functions. It is reported that GMSCs could suppress the proliferation of lymphocytes in peripheral blood. Under the stimulation of IFN-γ, GMSCs increased the expression of anti-inflammatory factors, such as IL-10, IDO, inducible NO synthase (iNOs), and cyclooxygenase 2 (COX-2). In vivo experiments using a colitis mouse model showed that the systemic infusion of GMSCs significantly ameliorated the pathological conditions. GMSC-treated mice displayed milder colitis activity, the restoration of injured intestinal sites, and reversed diarrhea and bodyweight [92]. Nakao et al., showed that GMSC-derived exosomes exert promising effects in periodontitis treatment. The periodontal bone resorption and the number of osteoclasts were attenuated. Treating GMSCs with TNF-α could increase the secreted exosomes, strengthen the positive effects, and enhance the polarization of anti-inflammatory M2 macrophages [93]. Another study showed the therapeutic application of GMSCs in collagen-induced arthritis, which resulted from the induction of T cell apoptosis via the FasL/Fas pathway [94].

### 5.4. Periodontal Ligament Stem Cells (PDLSCs)

The periodontal ligament (PDL) attaches dental roots to the alveolar bone. The PDL acquires a high alteration potential and contains PDLSCs. PDLSCs can give rise to osteo lineage cells, cementoblast-like cells, adipocytes, and chondrocytes. In vitro culture of PDLSCs forms a cementum/PDL-like structure, and periodontal-like tissues can be formed in vivo. PDLSCs are also the main participants in the repair of periodontitis lesions [59]. Liu et al., used membrane materials to transplant PDLSCs into periodontal defects in rats. The PDLSC-treated group showed a higher bone volume and trabecular thickness with the formation of cementum-like structures [95]. Moreover, PDLSCs could actively regulate the polarization of macrophages to create a suitable microenvironment for periodontal regeneration. Periodontal injury with PDLSC transplantation displayed more CD163^+^ cell infiltration and a higher expression of M2 macrophage markers, indicating that PDLSCs enhance the polarization of macrophage toward the anti-inflammatory M2 phenotype. In vitro experiments using the conditioned medium of PDLSCs (PDLSC-CM) to treat macrophages also confirmed the in vivo findings. M0 macrophages incubated with PDLSC-CM led to increased CD209^+^ and CD206^+^ M2 macrophages [96]. A higher expression of anti-inflammatory factors, including IL-10, TGF-β, and CCL18, was observed in the PDLSC-CM-treated group, while the pro-inflammatory molecule TNF-α was downregulated [95,96].

## 6. MSC-Based Treatment for Apical Periodontitis

### 6.1. MSC-Based Regenerative Endodontics

The common treatment for permanent teeth with apical periodontitis involves root canal therapy and apical surgery [97]. These classic treatment options aim to prepare, disinfect, and fill the root canal space to control infection and inflammation. The prognosis depends on whether the surrounding apical tissues could gradually repair and rebuild back to normal. Although root canal therapy has a high success rate and prolongs tooth survival, the non-vital pulp, the fragile tooth structure, and the loss of surrounding tissues may still accelerate tooth loss [98]. Therefore, biologically driven therapeutic strategies in endodontics have potential advantages. Stem-cell-based regenerative endodontic procedures are usually applied to AP-affected immature teeth. It is reported that the evoked-bleeding technique can also bring MSCs into the root canal in mature teeth with AP. In comparison with systemic blood, blood samples collected from AP lesions by the evoked-bleeding step showed higher expressions of MSC markers, including CD73, CD90, CD105, and CD146. Immunofluorescence experiments revealed that MSCs colocalized with the vascular structure in periapical lesions. This clinical study supported the potential of applying treatment strategies involving RET and periapical MSCs to mature teeth affected by AP [99]. However, there are still several problems with applying the apical bleeding technique in mature teeth compared with immature ones. First, the sources of MSCs are reduced since the apical papilla disappears in mature teeth. Second, the loss of the apical papilla and the narrower apical foramen restrict the blood flow volume. Third, the complex root canal system not only brings greater challenges for disinfection but also increases the difficulties for cell migration [100]. These features may attenuate the therapeutic effect of the evoked-bleeding treatment in mature teeth. Therefore, a recent study applied allogeneic MSCs in regenerative endodontics to treat AP patients.

Cordero et al., reported a clinical case using an allogeneic umbilical cord mesenchymal stem cells (UC-MSCs) encapsulated bio-scaffold to treat a complex AP patient. A 19-year-old male patient was diagnosed with AP during previous treatment, associated with cervical perforation. He was also undergoing orthodontic treatment. After routine treatment procedures, including the preparation and disinfection of the root canal and sealing of the perforation, allogeneic UC-MSCs were incorporated into platelet-poor plasma and implanted into the root canal. The follow-up examinations showed the recovery of the peri-radicular lesion and normal responses to percussion and palpation, accompanied by a response to the electric pulp test [101]. Recently, Gomez-Sosa et al., used cryopreserved allogeneic bone marrow MSCs for RET transplantation. A 55-year-old female patient suffered from swelling and sinus tract although she had already received root canal treatment and apical resection surgery. Unlike the former case, in this case, allogeneic BMSCs were cultured in vitro and encapsulated in pre-clotted platelet-rich plasma for transplantation. After 14 months of MSC implantation, a radiographic examination showed the healing of the apical lesion, apex remodeling, and increased periapical bone density. The treated tooth also presented sensitivity to cold and electric pulp tests [102]. These two cases both attained favorable clinical and radiographic outcomes of periapical tissues, suggesting the potential of allogeneic MSCs in regenerative endodontics and AP treatment. Nevertheless, the biggest flaws of allogeneic biomaterials are problems of safety, immunity, and ethics. Long-term follow-up tracing and examination are necessary. Compared with MSCs from other sources, dental MSCs have certain advantages when applied in RET and AP treatment. (1) Many lineages of dental MSCs are easily accessed. SHEDs are isolated from primary teeth by a minimally invasive procedure [58]. The third molar extraction surgery is another approach to obtaining dental MSCs and does not unnecessarily harm the patient. DPSCs, PDLSCs, and DFPCs can be separated from the third molars. Alveolar bone and gingival samples can also be collected for MSC culture during extraction surgery of impacted teeth. Moreover, the hPCy–MSC population is derived from conventional biological waste. Thus, tissue engineering using autologous dental MSCs is not restricted by access to stem cells. (2) Dental MSCs are naturally more suitable owing to the special embryonic origin of orofacial bone and proximate microenvironment [103]. (3) Dental MSCs exert promising differentiation capabilities for RET and AP treatment. DPSCs, DFPCs, PDLSCs, and SHEDs all acquire osteogenic and neurogenic potentials. PDLSCs can generate extra cementum, while SCAPs and DPSCs can differentiate into odontoblasts [5,82]. Therefore, we are looking forward to related research applying RET based on autologous dental MSCs.

### 6.2. Treatment Based on Extracellular Vesicles

Extracellular vesicles (Evs) are lipid membranous nanoparticles secreted by most cell types and carry different types of molecules. The EV cargo includes proteins, lipids, nucleic acids, glycans, etc., which reflect the features and activities of the original cells [104]. It is reported that a higher expression of CD63, an EV marker, was detected in the inflamed apical papilla area, suggesting the essential role of Evs in AP. Evs from LPS-treated dental pulp cells suppressed the odontoblastic ability of SCAPs by downregulating nuclear factor I/C (NFIC), an essential transcription factor for osteogenesis and tooth development. Afterward, Yang et al., developed NFIC-encapsulated Evs using HEK293FT cells, which could promote the proliferation, migration, dentinogenesis, and NFIC expression of SCAPs. Moreover, the NFIC-EV can be loaded on a gelatin scaffold along with SCAPs. This biomaterial displayed promising odontogenesis ability after being subcutaneously transplanted in nude mice. Increasing numbers of DMP-1-positive cells and collagen volume fraction were detected in SCAP-seeded scaffolds with NFIC-Evs [105]. Evs are essential components for cellular communication and promising candidates for targeted therapy. The lipid membrane of Evs can not only protect the cargo but also deliver it to the target cell with high efficiency. Using a cell line to generate Evs as a carrier to deliver biomolecules is a novel treatment option. Dental MSCs can be both the sources of Evs and the targets of EV-based therapy. Evs can also be loaded in biomaterials to facilitate the effects of stem-cell-based tissue engineering.

### 6.3. The Aid of Dentine Extracellular Matrix Proteins in MSC-Based Therapies

The regenerative activities led by MSCs require various growth factors, cytokines, chemokines, etc. For example, TGF-β, the bone morphogenetic protein (BMP) family, the vascular endothelial growth factor (VEGF) family, and the insulin growth factor (IGF) family coordinate the AP regeneration process [106,107,108]. However, these biomolecules tend to rapidly degrade after being secreted into the extracellular environment. It is reported that abundant biomolecules termed dentine extracellular matrix components (dECMs) can be sequestered within the dentine extracellular matrix during the dentinogenesis of odontoblasts. Notably, the bioactivity of dECMs remains highly preserved within the dentine matrix [109]. Research has shown that many therapeutic processes achieve the release of dECMs, including the application of root canal irrigation, pulp capping materials, epigenetic modifiers, and dental adhesive solution [110,111,112,113,114,115,116]. According to the principles of cell-free homing techniques, MSCs are recruited to the lesion site and activated in situ by supplying signaling molecules [117]. Soluble dECMs have shown a potent capacity to promote regeneration activities within several odontogenic MSC niches [118,119]. dECMs are promising agents in MSC-based therapies for AP.

The use of 17% EDTA in the chemo-mechanical debridement of root canal treatment can reportedly assist in and maximize the release of dECMs. However, the NaOCl solution negatively affects this observation [110,120]. These features of different irrigation solutions should be studied to balance the antiseptic ability and the capacity to promote dECMs exposure. Research has shown that ultrasonic agitation significantly promotes dECM release. These soluble biomolecules could then be delivered into the periapical area by the filing and preparation of the apical foramen [117]. Intracanal medicament using calcium hydroxide can not only function as an antimicrobial medicine but also prolong the favorable effects of dECMs [113,114]. Therefore, operations to enhance the generation of endogenous dECM components should be undertaken during root canal treatment. These approaches provide clinicians with a new angle of biologically driven therapy. These findings also inspire research on biomaterials and tissue engineering since promoting dECM exposure can be a novel target. Platelet-rich plasma is a widely used biologic that is rich in TGF-β, IGF1, platelet-derived growth factor (PDGF), and other growth factors [108,121]. In one study, the combination of a tricalcium phosphate (TCP) material and platelet-rich plasma showed a promising therapeutic effect in the treatment of AP lesions [108]. Although this case report did not focus on periapical MSCs, it showed that biomolecule-integrated materials provide many advantages in the treatment of AP with large bone lesions. A therapeutic approach focusing on upregulating the intrinsic regenerative ability of periapical MSCs needs to be further studied in the future.

## 7. Conclusions

Resulting from caries and pulp necrosis, AP is a common disease of the periapical area resulting from bacterial infection. The regeneration of periapical tissues, especially the destructive alveolar bone, has long been a difficult task in clinical treatment. The progression and repair processes of AP are related to the interactive regulatory network among pathogens, the immune system, and MSCs. Destructive and protective bioactivities take place simultaneously in AP. MSCs display a crucial function in the protective and healing process. They not only directly participate in the regeneration of inflamed periapical tissues but also have an immunoregulatory function to modulate the intensity of the immune reactions as well as the progression path of AP. The understanding of detailed mechanisms related to the dialogue between MSCs and immune components or mediators will boost the application of MSCs in inflammatory diseases. Remarkably, there have been developments in regenerative endodontics and MSC-targeted therapy that shed light on the treatment and prognosis of AP. However, the studies related to MSCs in AP are still in the early stages. The role of diverse lineages of MSCs in different stages of AP remains to be further explored. Although MSCs derived from periapical lesions have been characterized as a unique population, the sources of these MSCs remain to be elucidated. It is not clear which stem cell niche is activated by AP and participates in the rescue process. The development of single-cell RNA sequencing and lineage tracing technologies using transgenic mice will greatly contribute to the related research. Although MSC-based regenerative medicine has been applied in several studies on treating AP, many aspects, including the indications, the route of delivery, safety, dose, and frequency, remain to be explored. A deeper understanding of mechanisms underlying MSCs’ protective action will help to enhance stem-cell-based regenerative therapy for AP and other orofacial bone-related inflammatory diseases.

## Data Availability

Not applicable.
